# Complexity of the Basic Reproduction Number (R_0_)

**DOI:** 10.3201/eid2501.171901

**Published:** 2019-01

**Authors:** Paul L. Delamater, Erica J. Street, Timothy F. Leslie, Y. Tony Yang, Kathryn H. Jacobsen

**Affiliations:** University of North Carolina at Chapel Hill, Chapel Hill, North Carolina, USA (P.L. Delamater);; George Mason University, Fairfax, Virginia, USA (E.J. Street, T.F. Leslie, K.H. Jacobsen);; George Washington University, Washington, DC, USA (Y.T. Yang)

**Keywords:** basic reproduction number, theoretical models, disease outbreaks, infectious disease transmission, R_0_, mathematical modeling, outbreaks, basic reproduction ratio, basic reproduction rate, basic reproductive rate

## Abstract

The basic reproduction number (R_0_), also called the basic reproduction ratio or rate or the basic reproductive rate, is an epidemiologic metric used to describe the contagiousness or transmissibility of infectious agents. R_0_ is affected by numerous biological, sociobehavioral, and environmental factors that govern pathogen transmission and, therefore, is usually estimated with various types of complex mathematical models, which make R_0_ easily misrepresented, misinterpreted, and misapplied. R_0_ is not a biological constant for a pathogen, a rate over time, or a measure of disease severity, and R_0_ cannot be modified through vaccination campaigns. R_0_ is rarely measured directly, and modeled R_0_ values are dependent on model structures and assumptions. Some R_0_ values reported in the scientific literature are likely obsolete. R_0_ must be estimated, reported, and applied with great caution because this basic metric is far from simple.

The basic reproduction number (R_0_), pronounced “R naught,” is intended to be an indicator of the contagiousness or transmissibility of infectious and parasitic agents. R_0_ is often encountered in the epidemiology and public health literature and can also be found in the popular press (*1*–*6*). R_0_ has been described as being one of the fundamental and most often used metrics for the study of infectious disease dynamics (*7*–*12*). An R_0_ for an infectious disease event is generally reported as a single numeric value or low–high range, and the interpretation is typically presented as straightforward; an outbreak is expected to continue if R_0_ has a value >1 and to end if R_0_ is <1 (*13*). The potential size of an outbreak or epidemic often is based on the magnitude of the R_0_ value for that event (*10*), and R_0_ can be used to estimate the proportion of the population that must be vaccinated to eliminate an infection from that population (*14*,*15*). R_0_ values have been published for measles, polio, influenza, Ebola virus disease, HIV disease, a diversity of vectorborne infectious diseases, and many other communicable diseases (*14*,*16*–*18*).

The concept of R_0_ was first introduced in the field of demography (*9*), where this metric was used to count offspring. When R_0_ was adopted for use by epidemiologists, the objects being counted were infective cases (*19*). Numerous definitions for R_0_ have been proposed. Although the basic conceptual framework is similar for each, the operational definitions are not always identical. Dietz states that R_0_ is “the number of secondary cases one case would produce in a completely susceptible population” (*19*). Fine supplements this definition with the description “average number of secondary cases” (*17*). Diekmann and colleagues use the description “expected number of secondary cases” and provide additional specificity to the terminology regarding a single case (*13*).

In the hands of experts, R_0_ can be a valuable concept. However, the process of defining, calculating, interpreting, and applying R_0_ is far from straightforward. The simplicity of an R_0_ value and its corresponding interpretation in relation to infectious disease dynamics masks the complicated nature of this metric. Although R_0_ is a biological reality, this value is usually estimated with complex mathematical models developed using various sets of assumptions. The interpretation of R_0_ estimates derived from different models requires an understanding of the models’ structures, inputs, and interactions. Because many researchers using R_0_ have not been trained in sophisticated mathematical techniques, R_0_ is easily subject to misrepresentation, misinterpretation, and misapplication. Notable examples include incorrectly defining R_0_ (*1*) and misinterpreting the effects of vaccination on R_0_ (*3*). Further, many past lessons regarding this metric appear to have been lost or overlooked over time. Therefore, a review of the concept of R_0_ is needed, given the increased attention this metric receives in the academic literature (*20*). In this article, we address misconceptions about R_0_ that have proliferated as this metric has become more frequently used outside of the realm of mathematical biology and theoretic epidemiology, and we recommend that R_0_ be applied and discussed with caution.

## Variations in R_0_

For any given infectious agent, the scientific literature might present numerous different R_0_ values. Estimations of the R_0_ value are often calculated as a function of 3 primary parameters—the duration of contagiousness after a person becomes infected, the likelihood of infection per contact between a susceptible person and an infectious person or vector, and the contact rate—along with additional parameters that can be added to describe more complex cycles of transmission (*19*). Further, the epidemiologic triad (agent, host, and environmental factors) sometimes provides inspiration for adding parameters related to the availability of public health resources, the policy environment, various aspects of the built environment, and other factors that influence transmission dynamics and, thus, are relevant for the estimation of R_0_ values (*21*). Yet, even if the infectiousness of a pathogen (that is, the likelihood of infection occurring after an effective contact event has occurred) and the duration of contagiousness are biological constants, R_0_ will fluctuate if the rate of human–human or human–vector interactions varies over time or space. Limited evidence supports the applicability of R_0_ outside the region where the value was calculated (*20*). Any factor having the potential to influence the contact rate, including population density (e.g., rural vs. urban), social organization (e.g., integrated vs. segregated), and seasonality (e.g., wet vs. rainy season for vectorborne infections), will ultimately affect R_0_. Because R_0_ is a function of the effective contact rate, the value of R_0_ is a function of human social behavior and organization, as well as the innate biological characteristics of particular pathogens. More than 20 different R_0_ values (range 5.4–18) were reported for measles in a variety of study areas and periods (*22*), and a review in 2017 identified feasible measles R_0_ values of 3.7–203.3 (*23*). This wide range highlights the potential variability in the value of R_0_ for an infectious disease event on the basis of local sociobehavioral and environmental circumstances.

## Various Names for R_0_

Inconsistency in the name and definition of R_0_ has potentially been a cause for misunderstanding the meaning of R_0_. R_0_ was originally called the basic case reproduction rate when George MacDonald introduced the concept into the epidemiology literature in the 1950s (*17*,*19*,*24*,*25*). Although MacDonald used Z_0_ to represent the metric, the current symbolic representation (R_0_) appears to have remained largely consistent since that time. However, multiple variations of the name for the concept expressed by R_0_ have been used in the scientific literature, including the use of basic and case as the first word in the term, reproduction and reproductive for the second word, and number, ratio, and rate for the final part of the term (*13*). Although the frequent use of the term basic reproduction rate is in line with MacDonald’s original terminology (*9*), some users interpret the use of the word rate as suggesting a quantity having a unit with a per-time dimension (*7*). If R_0_ were a rate involving time, the metric would provide information about how quickly an epidemic will spread through a population. But R_0_ does not indicate whether new cases will occur within 24 hours after the initial case or months later, just as R_0_ does not indicate whether the disease produced by the infection is severe. Instead, R_0_ is most accurately described in terms of cases per case (*7*,*13*). Calling R_0_ a rate rather than a number or ratio might create some undue confusion about what the value represents.

## R_0_ and Vaccination Campaigns

Vaccination campaigns reduce the proportion of a population at risk for infection and have proven to be highly effective in mitigating future outbreaks (*26*). This conclusion is sometimes used to suggest that an aim of vaccination campaigns is to remove susceptible members of the population to reduce the R_0_ for the event to <1. Although the removal of susceptible members from the population will affect infection transmission by reducing the number of effective contacts between infectious and susceptible persons, this activity will technically not reduce the R_0_ value because the definition of R_0_ includes the assumption of a completely susceptible population. When examining the effect of vaccination, the more appropriate metric to use is the effective reproduction number (*R*), which is similar to R_0_ but does not assume complete susceptibility of the population and, therefore, can be estimated with populations having immune members (*16*,*20*,*27*). Efforts aimed at reducing the number of susceptible persons within a population through vaccination would result in a reduction of the *R* value, rather than R_0_ value. In this scenario, vaccination could potentially end an epidemic, if *R* can be reduced to a value <1 (*16*,*27*,*28*). The effective reproduction number can also be specified at a particular time *t*, presented as *R*(*t*) or *R_t_*, which can be used to trace changes in *R* as the number of susceptible members in a population is reduced (*29*,*30*). When the goal is to measure the effectiveness of vaccination campaigns or other public health interventions, R_0_ is not necessarily the best metric (*10*,*20*).

## Measuring and Estimating R_0_

Counting the number of cases of infection during an epidemic can be extremely difficult, even when public health officials use active surveillance and contact tracing to attempt to locate all infected persons. Although measuring the true R_0_ value is possible during an outbreak of a newly emerging infectious pathogen that is spreading through a wholly susceptible population, rarely are there sufficient data collection systems in place to capture the early stages of an outbreak when R_0_ might be measured most accurately. As a result, R_0_ is nearly always estimated retrospectively from seroepidemiologic data or by using theoretical mathematical models (*31*). Data-driven approaches include the use of the number of susceptible persons at endemic equilibrium, average age at infection, final size equation, and intrinsic growth rate (*10*). When mathematical models are used, R_0_ values are often estimated by using ordinary differential equations (*8*–*10*,*19*,*31*), but high-quality data are rarely available for all components of the model. The estimated values of R_0_ generated by mathematical models are dependent on numerous decisions made by the modeler (*8*,*32*,*33*). The population structure of the model, such as the susceptible-infectious-recovered model or susceptible-exposed-infectious-recovered model, which includes compartments for persons who are exposed but not yet infectious, as well as assumptions about demographic dynamics (e.g., births, deaths, and migration over time), are critical model parameters. Population mixing and contact patterns must also be considered; for example, for homogeneous mixing, all population members are equally likely to come into contact with one another, and for heterogeneous mixing, variation in contact patterns are present among age subgroups or geographic regions. Other decisions include whether to use a deterministic (yielding the same outcomes each time the model is run) or stochastic (generating a distribution of likely outcomes on the basis of variations in the inputs) approach and which distributions (e.g., Gaussian or uniform distributions) to use to describe the probable values of parameters, such as effective contact rates and duration of contagiousness. Furthermore, many of the parameters included in the models used to estimate R_0_ are merely educated guesses; the true values are often unknown or difficult or impossible to measure directly (*31*,*34*,*35*). This limitation is compounded as models become more complex and, thus, require more input parameters (*20*,*35*), such as when using models to estimate the value of R_0_ for infectious pathogens with more complex transmission pathways, which can include vectorborne infectious agents or those with environmental or wildlife reservoirs. In summary, although only 1 true R_0_ value exists for an infectious disease event occurring in a particular place at a particular time, models that have minor differences in structure and assumptions might produce different estimates of that value, even when using the same epidemiologic data as inputs (*20*,*31*,*32*,*36*,*37*).

## Obsolete R_0_ Values

New estimates of R_0_ have been produced for infectious disease events that occurred in recent history, such as the West Africa Ebola outbreak (*34*,*38*,*39*). However, for many vaccine-preventable diseases, the scientific literature reports R_0_ values calculated much further back in history. For example, the oft-reported measles R_0_ values of 12–18 are based on data acquired during 1912–1928 in the United States (R_0_ of 12.5) and 1944–1979 in England and Wales (R_0_ of 13.7–18.0) (*14*), even though more recent estimates of the R_0_ for measles highlight a much greater numeric range and variation across settings (*23*). For pertussis (R_0_ of 12–17), the original data sources are 1908–1917 in the United States (R_0_ of 12.2) and 1944–1979 in England and Wales (R_0_ of 14.3–17.1) (*14*). The major changes that have occurred in how humans organize themselves both socially and geographically make these historic values extremely unlikely to match present day epidemiologic realities. Behavioral changes undoubtedly have altered contact rates, which are a key component of R_0_ calculations. Yet, these R_0_ values have been repeated so often in the literature that newer R_0_ values generated by using modern data might be dismissed if they fall outside the range of previous estimates. Given that R_0_ is often considered when designing and implementing vaccination strategies and other public health interventions, the use of R_0_ values derived from older data is likely inappropriate (*23*). Decisions about public health practice should be made with contemporaneous R_0_ values or *R* values instead.

## Conclusions

Although R_0_ might appear to be a simple measure that can be used to determine infectious disease transmission dynamics and the threats that new outbreaks pose to the public health, the definition, calculation, and interpretation of R_0_ are anything but simple. R_0_ remains a valuable epidemiologic concept, but the expanded use of R_0_ in both the scientific literature and the popular press appears to have enabled some misunderstandings to propagate. R_0_ is an estimate of contagiousness that is a function of human behavior and biological characteristics of pathogens. R_0_ is not a measure of the severity of an infectious disease or the rapidity of a pathogen’s spread through a population. R_0_ values are nearly always estimated from mathematical models, and the estimated values are dependent on numerous decisions made in the modeling process. The contagiousness of different historic, emerging, and reemerging infectious agents cannot be fairly compared without recalculating R_0_ with the same modeling assumptions. Some of the R_0_ values commonly reported in the literature for past epidemics might not be valid for outbreaks of the same infectious disease today.

R_0_ can be misrepresented, misinterpreted, and misapplied in a variety of ways that distort the metric’s true meaning and value. Because of these various sources of confusion, R_0_ must be applied and discussed with caution in research and practice. This epidemiologic construct will only remain valuable and relevant when used and interpreted correctly.
